# Perceived Support Is Not Psychological Change: Reframing AI Chatbots in Mental Health Care

**DOI:** 10.2196/94503

**Published:** 2026-08-26

**Authors:** Michal Mahat-Shamir, Maya Payes, Maya Kagan

**Affiliations:** 1 School of Social Work, Ariel University, 3 Kiryat HaMada, Ariel, Judea and Samaria Area, 40700, Israel, 972 0506211188

**Keywords:** generative artificial intelligence, digital mental health, therapeutic alliance, psychological change, digital literacy

## Abstract

AI chatbots are increasingly used for emotional support, often outside formal care. Emerging evidence suggests that generative systems may produce modest reductions in symptoms of depression and anxiety. Yet a change in symptoms alone does not clarify the nature of the process involved. This viewpoint argues that chatbot interactions may primarily operate through structured mirroring and self-reflection rather than through the intersubjective engagement that underpins therapeutic transformation. We propose an implementation-focused distinction between tools that support symptom regulation and engagement and interventions that aim at enduring psychological change, emphasizing the need to specify mechanisms, relational conditions, and appropriate care pathways. Conflating perceived support with psychological change risks redefining therapeutic standards around technological affordance rather than relational encounters. As AI chatbots become embedded in mental health ecosystems, conceptual clarity and digital emotional literacy are essential to ensure that innovation strengthens rather than replaces the relational foundations of care.

## Context and Current Evidence

Across the world, people are increasingly turning to AI chatbots for emotional support. In this paper, we use the term AI chatbots consistently to refer to generative AI–based conversational systems used in mental health contexts. Empirical evidence indicates that users engage with these systems not only for information or problem-solving, but because the interaction feels emotionally supportive, reducing distress and creating a sense of being heard [1]. This pattern aligns with longstanding findings that individuals respond to conversational technologies as if they were social partners rather than mere tools [2,3]. Such use often occurs outside formal care, without clinical oversight.

At the same time, the clinical ecosystem is rapidly exploring AI chatbots for mental health, alongside calls to translate large language models responsibly into care contexts [4,5]. Systematic reviews have begun mapping both the promise and limitations of AI chatbots in mental health, including variability in evidence quality, safety concerns, and limited long-term outcome data [6]. Recent meta-analytic findings suggest that AI chatbots are associated with modest but statistically significant reductions in symptoms of depression and anxiety [7]. Earlier controlled trials of rule-based AI chatbots similarly reported feasibility, engagement, and short-term symptom improvement in selected populations [1].

These findings have encouraged a pragmatic, outcomes-first stance: if symptom scores improve, clinical deployment may appear justified.

To clarify the conceptual distinctions that guide this paper, we use the term *perceived support* to refer to the subjective experience of being heard, understood, or emotionally validated within an interaction, regardless of the source of that interaction. *Symptom regulation* refers to short-term changes in affective or cognitive states, such as reductions in distress, anxiety, or depressive symptoms. By contrast, *psychological change* denotes more enduring transformations in how individuals experience themselves, others, and their internal worlds, typically involving shifts in meaning, relational patterns, or self-structure. While these processes may co-occur, they are not equivalent. The distinction between them is central to the present argument, which examines whether AI chatbot interactions primarily support perceived support and symptom regulation, or whether they can also facilitate deeper forms of psychological change.

## Outcomes Versus Mechanisms in Digital Mental Health

Yet this evaluation rests on an implicit assumption that warrants scrutiny: that symptom reduction is equivalent to therapeutic change. Even when measurable improvement is observed, a more fundamental question remains insufficiently examined: what psychological process underlies such change?

Implementation science emphasizes that digital interventions should specify mechanisms of action and the conditions under which benefits occur [4-6]. Outcome metrics alone do not clarify whether change emerges through expectancy effects, structured cognitive rehearsal, psychoeducation, or short-term emotional regulation. Future research may benefit from complementing symptom-based outcome measures with approaches that assess meaning reconstruction, relational functioning, and changes in self-experience over time. Such work could combine validated psychometric instruments with qualitative longitudinal designs capable of capturing shifts in narrative identity, interpersonal patterns, and subjective understandings of change. A broader measurement framework may help distinguish symptom regulation from more enduring forms of psychological transformation. This distinction is particularly important in light of recent meta-analytic findings showing modest but statistically significant reductions in symptoms associated with chatbot use, without clear identification of the mechanisms through which such changes occur [6,7]. From a theoretical perspective, this gap underscores the need to differentiate between observable outcomes and the underlying processes that generate them, a distinction that has long been emphasized in psychotherapy research. At the same time, it is possible that symptom improvement and perceived support may, in some cases, serve as a pathway toward broader psychological change, particularly when they enable engagement, reflection, or help-seeking. However, such pathways are likely to be contingent on contextual factors, including the nature of the system, the user’s needs, and the presence of broader relational or clinical supports, rather than arising from chatbot interaction alone.

Reviews of the digital therapeutic alliance indicate that alliance-like constructs may emerge in technology-mediated interventions, but their structure, measurement, and predictive value may differ from face-to-face psychotherapy [8,9]. An outcomes-first argument is understandable in contexts of clinician scarcity and unmet need. However, implementation decisions require more than effect sizes. They require clarity about mechanisms, appropriate indications, risk thresholds, and integration within care pathways. Without such distinctions, the field risks conflating perceived support with psychological transformation.

## Relational Processes and Structured Mirroring

AI chatbots are not a homogeneous category. They vary considerably in their underlying architecture, level of generativity, domain specificity, and degree of clinical integration. The present argument, therefore, focuses on common interactional patterns observed across many contemporary systems, while recognizing that different implementations may afford distinct relational and psychological dynamics.

Against this backdrop, empirical research in human-AI interaction helps explain why chatbot exchanges can feel supportive. Users often disclose personal information more readily to digital agents, partly due to reduced fear of judgment and impression management [10]. Design features that simulate empathic responsiveness can increase perceived intimacy, satisfaction, and intention to reuse chatbot-based counseling [11].

These dynamics can be understood as reflecting a process of structured mirroring: users articulate concerns, and the system reflects, reframes, and validates those articulations fluently. Such mirroring can stabilize affect and facilitate self-reflection. These findings further indicate that responses perceived as empathic and reflective are associated with increased user disclosure, perceived understanding, and short-term emotional relief, even in the absence of a human interlocutor [10,11]. This suggests that the stabilizing effects described here may emerge through processes that resemble relational attunement, while remaining structurally grounded in patterned responsiveness rather than intersubjective engagement.

To make this process more concrete, it can be illustrated through a typical interaction pattern. For example, a user may express distress (eg, “I feel overwhelmed by my mother’s decline and don’t know how to cope”), to which the chatbot responds by reflecting and elaborating the user’s experience: “That sounds very heavy and painful. Watching a parent change can feel destabilizing, as if something fundamental is shifting.” The system may then organize the experience further by identifying themes (“It seems like you are holding both concern for her and fear about what this means for you”) and offering structured avenues for reflection (“Would you like to explore what feels most difficult right now, or how this affects you personally?”).

Such exchanges exemplify structured mirroring: the user’s internal experience is articulated, expanded, and organized, often in ways that enhance coherence and emotional regulation, without introducing an independent perspective that fundamentally alters the underlying relational or symbolic structure. This process typically operates within the user’s existing experiential and narrative framework, rather than introducing novel meaning from an external subject position. However, this form of structured mirroring differs from theoretical accounts of therapeutic transformation, which emphasize encounter with an “other” that exceeds the self’s existing narrative. Bakhtin described this as the “surplus of seeing,” the capacity of another subject to perceive what one cannot perceive in oneself [12]. It is this externality that enables new meanings to emerge.

Clinical formulations similarly describe transformation as arising within relational fields. Ogden’s concept of the “analytic third” refers to a co-created psychological space between therapist and patient in which new emotional configurations become possible [13]. Such accounts presuppose interaction between 2 subjectivities. AI chatbots, however sophisticated, do not occupy an independent subject position; they generate responses contingent on user input and training data.

From a Lacanian perspective, AI-mediated exchanges may intensify processes of mirroring associated with the Imaginary register of self-recognition and coherence [14,15]. This may explain why interactions feel containing and supportive. Yet deeper psychological transformation often involves negotiating difference, ambiguity, and symbolic disruption—processes that extend beyond structured reflection.

At the same time, it is important to acknowledge that human psychotherapy is not uniformly transformative. Many therapeutic approaches focus primarily on symptom reduction, and even relationally oriented treatments may involve misunderstandings, alliance ruptures, or limited clinical gains. The present argument, therefore, does not idealize human therapy but rather highlights that the possibility of intersubjective engagement, including the negotiation of difference and rupture, constitutes a distinctive feature of human therapeutic relationships, even when such processes do not always lead to lasting change.

## Clinical and Implementation Implications

The central practical question is not whether chatbots can provide help, but what kind of help they provide and how they should be positioned within care systems. Scoping and systematic reviews of AI chatbots in mental health describe applications including psychoeducation, coping support, cognitive behavioral therapy–informed exercises, and service navigation, with heterogeneous evidence quality and population fit [7,16]. Taken together, these findings suggest that chatbot-based interventions primarily support engagement, coping, and short-term symptom relief, aligning with the distinction proposed in the present paper between processes that regulate experience and those that enable deeper psychological transformation. Accordingly, these findings support a differentiated implementation model. Chatbots may serve as low-intensity supports for articulation and skills practice, adjunct tools that enhance engagement between sessions, or triage mechanisms that direct users toward human care when risk, complexity, or chronicity are present [4-6]. By contrast, presenting such systems as substitutes for psychotherapy risks institutionalizing interactions that simulate relational presence without providing intersubjective engagement. From a clinical and implementation perspective, these distinctions can be operationalized through several practical criteria for appropriate use. Chatbots may be most suitable in contexts characterized by low to moderate distress, where the primary goals are articulation, emotional regulation, or skills practice. By contrast, cases involving high clinical risk, significant relational complexity, or enduring patterns of distress may require human involvement, particularly where interpretation, rupture, or meaning-making are central to the therapeutic process. Indicators that chatbot use may be insufficient include escalating distress despite repeated use, increasing reliance on the system without functional improvement, or expressions suggesting risk, isolation, or impaired reality testing. Conversely, appropriate deployment may involve clearly bounded use as an adjunct or entry point within broader care pathways, rather than as a standalone substitute for relational treatment [4-6]. This approach aligns with emerging calls for stepped and integrated models of digital mental health care. These criteria are intended to support informed clinical judgment rather than prescriptive decision-making. At the same time, these recommendations must be considered within the realities of contemporary mental health systems, where long waitlists, workforce shortages, and limited access to care may constrain the availability of human alternatives. In such contexts, directing individuals toward human care raises practical and ethical tensions, particularly when recommended services are inaccessible. Recognizing these constraints does not negate the importance of human involvement in complex cases but rather underscores the need to view AI chatbots as components of broader stepped-care systems rather than as substitutes for investment in accessible mental health services [4-6].

These considerations are not merely theoretical. Clinical standards shape procurement, reimbursement, governance, and professional accountability. As digital ecosystems scale rapidly, conceptual slippage, mistaking reflective coherence for relational presence, may subtly redefine what counts as therapeutic engagement.

These considerations also raise important questions regarding risk and safety. While chatbot interactions may provide a sense of support and coherence, they may also foster unintended dependency or delay help-seeking. They may also reinforce maladaptive interpretations, particularly when users attribute greater understanding or intentionality to the system than is warranted. In addition, large language models are subject to technical limitations that may further amplify these risks. These include algorithmic hallucinations, the absence of ground-truth understanding, and a tendency to respond in ways that affirm user statements even when such validation may be inaccurate or clinically unhelpful. Such characteristics may inadvertently reinforce distorted beliefs or maladaptive interpretations, particularly among individuals experiencing severe distress or impaired reality testing [17]. These risks may be particularly salient among vulnerable populations, including individuals experiencing social isolation or difficulties in reality testing, for whom simulated responsiveness may be mistaken for relational presence.

Importantly, these vulnerabilities are unlikely to be distributed uniformly across users. The psychological impact of AI chatbots and the likelihood of mistaking simulated empathy for genuine connection may vary across developmental stages, cultural contexts, and levels of digital literacy. Such heterogeneity suggests that the meaning and consequences of AI-mediated support are likely to differ between individuals and should not be assumed to be universal [18]. In such contexts, reliance on chatbot interaction alone may obscure the need for human engagement, potentially prolonging distress or limiting access to appropriate care. These concerns should also be considered within the broader commercial context in which many AI chatbots are developed and deployed. Business models that prioritize user engagement and prolonged interaction may unintentionally create incentives that are not always aligned with clinical goals, particularly when dependency or continued use becomes commercially advantageous [17]. Recognizing these tensions highlights the importance of governance frameworks that place user well-being, transparency, and accountability above engagement metrics alone.

## Digital Emotional Literacy

The task is not to dismiss AI-mediated support, but to situate it accurately within mental health care. Conceptual clarity should be treated as a core competency for clinicians, developers, and policymakers: distinguishing perceived support and symptom regulation from relational processes intended to produce enduring psychological change.

Recent frameworks emphasize responsible translation of AI chatbots into care settings, including evaluation standards, safety oversight, and pathway integration [4,5]. Systematic reviews similarly highlight limitations in emotional understanding, safety validation, and longitudinal evidence [6]. At the same time, it is important to acknowledge that the rapid development of AI chatbots has outpaced the accumulation of robust empirical evidence regarding their long-term psychological impact. As such, several of the conceptual distinctions proposed here should be understood as provisional and open to revision as the evidence base evolves.

Importantly, the present argument should not be interpreted as evidence that AI chatbots are inherently incapable of facilitating enduring psychological change. Rather, we propose that their effects may currently be better understood in terms of structured mirroring and symptom regulation. Whether AI-mediated interactions can contribute to more enduring transformations in meaning, self-structure, or relational functioning remains an open empirical question that warrants future longitudinal investigation.

We propose digital emotional literacy as a practical orientation: an ability to recognize what AI chatbots can and cannot provide psychologically, to communicate those limits transparently, and to embed tools within care models that preserve relational foundations. Such literacy is not merely an individual attribute but also a systemic responsibility that can be fostered at the level of users, clinicians, and health care systems. In practical terms, digital emotional literacy may extend to public and patient education initiatives aimed at helping users understand both the potential benefits and the limitations of AI-mediated support, thereby fostering more informed and responsible engagement with these technologies [18]. It may also include incorporating AI-related competencies into the education and training of mental health professionals, enabling clinicians to discuss chatbot use with patients, address unrealistic expectations, and recognize situations in which referral to human care is warranted [17]. Finally, digital emotional literacy may involve the development of implementation guidelines that specify appropriate indications, risk thresholds, and safeguards for integrating AI chatbots into stepped-care systems [19]. Safeguarding this distinction is not an obstacle to innovation. It is a prerequisite for ensuring that digital mental health advances without eroding the relational conditions under which lasting psychological change is most likely to occur.

## Conclusion

AI chatbots may provide meaningful perceived support, facilitate self-reflection, and contribute to symptom regulation. However, perceived support should not automatically be equated with enduring psychological change. The distinction proposed in this paper is not intended to diminish the value of AI-mediated support, but to clarify the mechanisms through which benefits may occur and the contexts in which human relational processes remain essential. As evidence continues to evolve, future research should examine whether AI-mediated interactions can contribute to more enduring transformations in meaning, self-structure, and relational functioning. Until then, conceptual clarity and digital emotional literacy remain essential for integrating AI responsibly into mental health care.
